# Heat shock exposure during early wheat grain development can reduce maximum endosperm cell number but not necessarily final grain dry mass

**DOI:** 10.1371/journal.pone.0285218

**Published:** 2023-04-28

**Authors:** Christine Girousse

**Affiliations:** INRAE, UCA, UMR 1095 GDEC, 5 Chemin de Beaulieu, Clermont-Ferrand, France; University of Delhi, INDIA

## Abstract

Post-anthesis heat shocks, which are expected to increase in frequency under climate change, may affect wheat grain development and lead to significant decreases in grain yield. Grain development occurs in three phases, the lag-phase, the filling-phase, and maturation. The growth of the three main compartments of the grain (outer layers (OLs), endosperm, embryo) is staggered, so that heat shocks affect time- and tissue-specific growth processes differentially depending on their timing. We hypothesized that heat shocks during the lag-phase may reduce final grain size, resulting from a reduction in endosperm cell number and/or a restricted OLs growth. Plants were heated for four consecutive days during the lag-phase or the filling-phase or both phases (lag- and filling-). Heat shocks consisted in four hours a day at 38°C and 21°C for the rest of the day. Controlled plants were maintained at 21/14°C (day/night). For each temperature treatment, kinetics of whole grain and compartment masses and dimensions were measured as well as the endosperm cell number. An early heat shock reduced endosperm cell proliferation. However, the growth patterns neither of endosperm nor of OLs were modified compared to controls, resulting in no differences in final grain size. Furthermore, compared to controls, a single heat shock during the filling-phase reduced both the duration and rate of dry mass accumulation into grains, whereas two consecutive shocks reduced the duration but enhanced the rate of dry mass of accumulation, even when endosperm cell number was reduced. The mean endosperm cell size was shown to be larger after early heat shocks. All together, these results suggest a compensatory mechanism exists to regulate endosperm cell size and number. This process might be a new mechanistic target for molecular studies and would improve our understanding of post-anthesis wheat tolerance to heat-shocks.

## Introduction

Global agriculture has to face new challenges including climate changes. Model-based projections of climate change suggest in particular that the mean surface temperature over land regions will continue to increase by the end of the century, especially if greenhouse gas emissions continue to increase [1]. Moreover, this global warming is predicted to be accompanied by an increase of the frequency of heat waves [2–4]. Temperature increases have the most likely negative impact on crop [1, 5–7]. In particular, wheat (*Triticum aestivum* L.), a cool-season cereal with an optimum growing mean temperature ranging between 12°C and 24°C [8, 9], is very sensitive to temperature increases that likely prompt yield reductions in most wheat growing areas [10–14]. Asseng *et al*. (2015) [13] estimated that global wheat production would decrease by 6% for each °C of further temperature increase.

Reproductive stages of wheat development are the most sensitive to high temperatures [9, 15, 16]. Indeed, exposures to high temperatures occurring immediately around anthesis can significantly reduce grain number by increasing floret mortality and/or sterility [17, 18]. High temperatures occurring a few days after anthesis can lead to decreased grain individual mass [19, 20]; this is mainly the result of a reduced duration of grain growth, which is not always compensated by an increase in grain growth rate [21–23]. The magnitude of the impact of high temperatures on final grain individual mass depends upon the combination of their intensity and duration of exposure such that both longer exposure duration and/or higher temperature values are more damaging. In this regard, one must distinct chronic vs acute heat stress [24, 25]. Chronic high temperature stress referred to exposures of plants to sustained periods of moderately warmer than average temperature (around 3°C to 6 °C above ambient, ca. 25–32 °C mean day temperature); acute heat stress (or heat shock (HS)) referred to exposures of short periods to very high temperatures (ca 33–40°C). While the effects of chronic heat stress on wheat grain growth have been well studied for a long time [20, 21, 26–29], in the past there have been relatively few studies on the impact of acute heat stress [18, 19, 22, 30] until recently [31–33]. And this, despite the fact that acute heat stress might be more damaging for wheat grain final mass than would be expected from chronic heat stress [20, 30, 34, 35]. Then, our understanding of the effects of heat shocks on the physiological processes underlying the individual grain growth still needs studies.

Wheat grain is a complex organ composed of three major compartments: the outer layers, the starchy endosperm and the embryo, all including several tissues with different anatomical features and distinct functions. Final grain mass is highly correlated with the initial mass of the ovule mass prior fertilization [18, 36] and onwards results from various processes occurring in those compartments. From ovule fertilization onwards, whole grain growth and development is classically divided into three phases [37, 38], based on the processes occurring into the endosperm: lag-phase (from anthesis to around 10–15 days after anthesis (DAA)), filling-phase (from 15 to 35–40 DAA), and maturation phase. Grain development is initiated by ovule fertilization; the ovary epidermis and parenchyma give the grain pericarp tissues, while the endosperm and the embryo start to develop. During the lag-phase, cell proliferation occurs within the grain endosperm; water is rapidly accumulated inside the grain, contributing most to the volumetric growth of the grain [39, 40]. During this first phase, the outer layers (OLs) of the grain enlarge during a very short period of cell division followed by cell expansion [41], thereby constituting the main component of the wheat grain at that time [42]. OLs reached their maximum dry matter mass and water content before the end of the lag-phase and these traits are well correlated with the final grain mass [43]. Meanwhile, the embryo begins to grow and differentiate [44]. At the end of the lag-phase, the grain length is set [45, 46]; the maximum number of endosperm cells is attained and correlates positively with the grain-filling capacity and consequently with final individual grain mass [47]. The filling-phase consists of the accumulation of assimilates into the endosperm (mainly starch and proteins); the grain water mass remains constant and dry mass content increases. During this phase, the OLs dry mass decreases rapidly [43], resulting from programmed cell death especially in the mesocarp tissues [48]. The embryo reaches its maximum length [44]. During the maturation phase, grain desiccates because of water loss while, within the endosperm cells, the storage proteins polymerize and fit together with starch contributing to define grain quality. Thus, depending on the timing of their occurrence, high temperatures will affect differentially specific growth processes within the grain [32]; this is particularly true when considering heat shocks that expose plants to high temperatures for short durations.

Numerous studies have demonstrated that exposures to heat shocks during the filling-phase of grain development (from 15 to 35 DAA) affect the final individual grain mass. It is mainly achieved through a reduction of the duration of dry mass accumulation associated with reduced activity of the soluble starch synthesis (SSS) activity and through a concomitant reduction of water content and volume expansion into the grain [19, 20, 22, 29, 32, 33, 49, 50]. Then, the reduction of dry mass accumulation into the grain following exposure to heat shocks is unlikely due to a limitation of assimilates supply to the grain [32, 33, 49, 51], even though there is a large genotypic variability associated with the response of wheat grain growth in response to heat shocks [28, 31, 52]. In contrast, heat shock exposures during the lag-phase of grain development (from 3 DAA to 10 DAA) have received less attention. Regarding the processes involved in the grain development during the lag-phase, early heat shocks might affect the final individual grain mass mainly through a reduction of the rate or duration of (i) the cell multiplication in the endosperm or (ii) of the pericarp expansion as both of these processes are well associated with the final grain mass [43, 47]. Some studies seem to contradict the first proposition: for example, Wardlaw (1970) [26] found that the final cell number within the endosperm was independent of temperature, although the rate of cell division was greater at higher temperature. Similarly, Nicolas *et al*. (1984) [51] found no effect of high temperatures on endosperm maximum cell number or size. However, these authors exposed wheat plants during the first 3–10 DAA to moderately high temperatures (27/22°C [26] or 28/20°C [51]) and not to heat shocks. To our knowledge, the impact of heat shocks on pericarp growth during the lag-phase has never been considered. Moreover, the effects of post-anthesis consecutive heat shocks on grain development received less attention, despite an increasing frequency of heat waves as predicted by climate models [4].,.

Therefore, the present experiment was undertaken with the aim to (i) study the impact of heat shocks during the lag-phase of grain growth (5 DAA) on some of the main processes involved in the final grain size and mass, namely, grain and OLs fresh and dry masses and water accumulation, grain volume expansion and endosperm cell proliferation; (ii) compare the effect of heat shocks occurring during the lag- or the filling- phases on the same processes and (iii) determine whether or not the effects of two consecutive heat shocks are additive; if this were the case, it would simplify predictions of the effects of two consecutive heat shocks and then contribute to improve current crop models.

## Materials and methods

### Plant material and culture conditions

Crops of the winter wheat (*Triticum aestivum* L.) cultivar Récital were grown outside at INRAE Clermont-Ferrand, France (45°46’ N, 03°09’ E, 329 m a.s.l.), in eight 2.0-m^2^ containers, 0.5 m deep, filled with a black soil-peat (2:1, v/v) mixture. Seeds were sown at a density of 500 seed m^-2^. The dense planting limited the development of axillary tillers and resulted in homogeneous canopies at anthesis. Nitrogen was supplied as ammonium-phosphate (N:P, 18:46); 5 g N m^-2^ was supplied about 1 week after the start of tillering, 10 g N m^-2^ when the stem started to elongate and 5 g N m^-2^ at anthesis. Weather conditions (mean daily temperature, rainfall and global solar radiation) from sowing to anthesis were accessed from measurements at the meteorological station of Aulnat (~1 km from the experimental site) and are presented on S1 Fig. From sowing to anthesis, the average daily temperature was 8°C with a minimum value of -8.3°C and a maximum value of 19.2°C. The average daily solar radiation was 8.3 MJ m^-2^. From sowing to anthesis, the crops received 221 mm of rainfall and 200 mm of watering to maintain the soil water content above 80% of field capacity.

At anthesis, we characterized the plant canopy of the eight containers by recording flowering dates and the number of spikelets per spike for 200 randomly selected spikes on the main stems; in addition, we measured the spike length and spike mass of 10 randomly selected spikes on the main stems. Then, four among the eight containers were selected for the homogeneity of plant canopy (S1 Table) and were transferred to transparent enclosures under natural light in the Crop Climate Control and Gas Exchange Measurement (C3-GEM) platform. The C3-GEM platform allows air temperature, air CO_2_ concentration, water supply and gas exchange to be monitored and controlled [23, 53]. The rate and frequency of watering were adjusted to maintain soil water potential above -0.3 MPa. Except during heat stress exposure, day/night air temperature was maintained at 21/14 ± 0.2°C. Day/night air relative humidity was maintained at 81.8/82.4 ± 7%, corresponding to day/night air vapor pressure deficit (VPD) of 0.45/0.28 ± 0.14 kPa. Air temperature next to wheat spikes was measured using copper-constantan thermocouples placed under a shield screen.

As air and grain temperature may differ from each other [20] and as grain temperature is more relevant than air temperature in terms of grain developmental processes, grain temperature was also measured using 0.2-mm copper-constantan thermocouples inserted into basal grains of central spikelets. As the insertion of the thermocouple into the grain caused local necrosis, thermocouples were moved to different grains every 2 days. Air and grain temperatures were recorded every 10 s on a CR1000 datalogger (Campbell Scientific Ltd, Shepshed, UK) and averaged over 10 min. Four air and four grain thermocouples were equally spaced in each container. After spike emergence, 200 main-stems with similar spikelet number per spike (18 or 19 spikelets per spike) were tagged in each container. Each tagged spike was labelled with the date of first anther extrusion on the central spikelet (that is, the ninth or tenth spikelets, with spikelets numbered from the base). From this day, the time-course of grain development was expressed in thermal time (°Cd, base 0°C) calculated using daily mean grain temperature [43, 45].

### Temperature treatment and heat shock application

From anthesis, four temperature treatments were imposed, each treatment being applied to one plant container. Plants of the control treatment (Control) were maintained from anthesis to maturity at 21°C (from 6:00 to 21:00 GMT) and 14°C (from 21:00 to 6:00 GMT); the average daily air temperature, relative humidity and VPD were 18.5°C, 82% and 0.39kPa respectively. Heat-stressed plants were heated for four consecutive days during (i) the lag-phase of grain development from ~90°Cd after anthesis (5 DAA; HS1); endosperm cell multiplication has been shown to occur at that time for Récital cv [46, 54]; (ii) the grain filling-phase from ~420°Cd after anthesis (23 DAA; HS2); or (iii) both the lag- and filling- phases, from ~90°Cd and ~420°Cd after anthesis respectively (HS12). The second heat shock was applied on the same grain developmental stage (420°Cd), expressed in thermal time whatever the heat treatment (HS2, HS12), independently of the putative effects of high temperatures on grain developmental timing under HS12 treatment. Heat shocks consisted in four hours a day at 38°C (air temperature), between 12:00 and 16:00, and 21°C (air temperature) for the rest of the day; the average daily air temperature was 23.8°C. The gradual rate of heating or cooling was 8.5°Cd h^-1^. During the four hours of heat shock, the air relative humidity ranged from 40% to 50% and the air VPD from 3.3 to 3.9 kPa. At high soil water potential, as in this study, leaf net CO_2_ assimilation rate for wheat does not respond to increased VPD [55], thus the higher VPD during the heat shocks should not have modified the whole plant response.

### Sampling procedures and grain measurements

**Fig 1:**
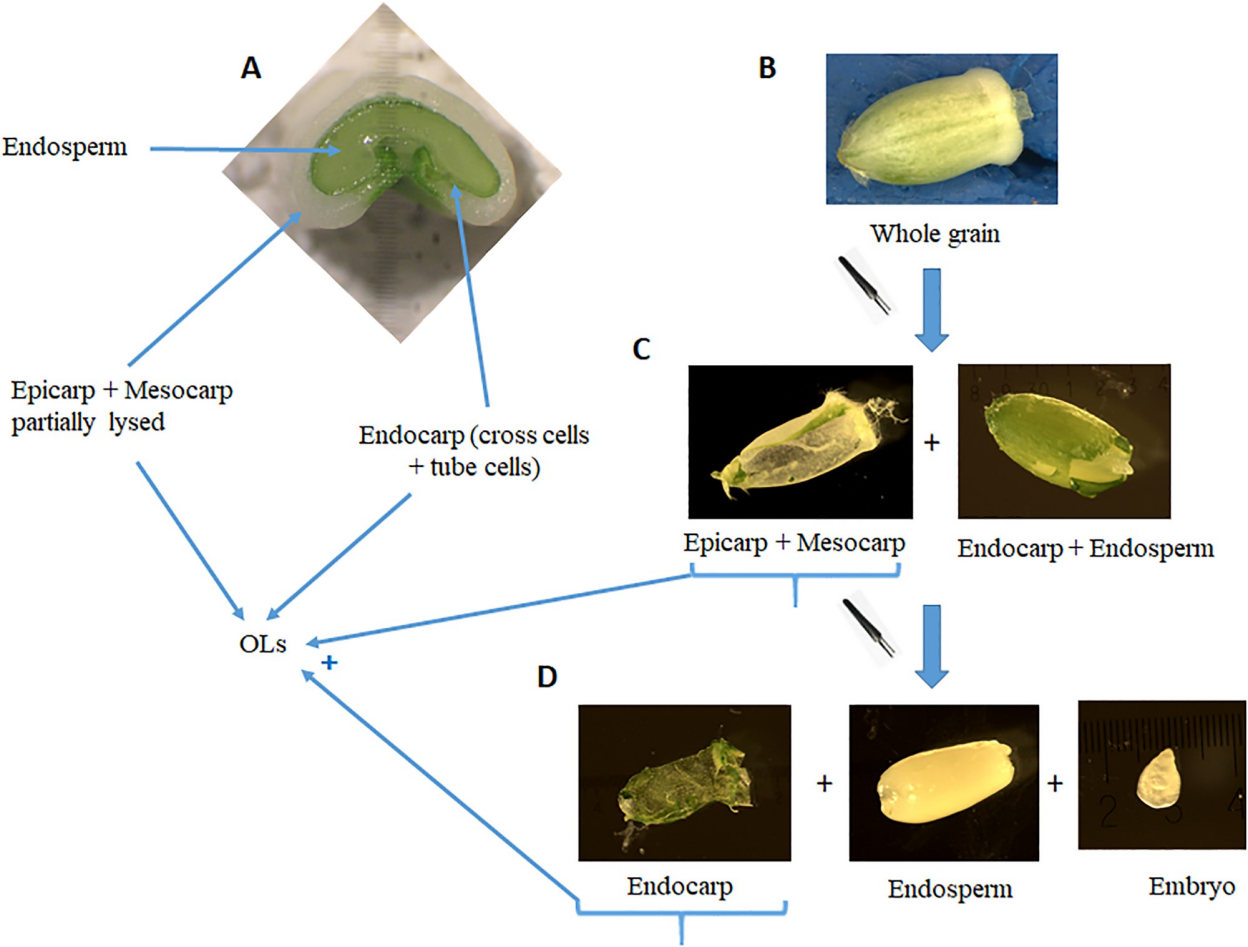
Pictures illustrating the dissected compartments. A representative fresh cross section of the grain (150°DAA) showing the main different tissues (A): endosperm is composed of testa + aleurone layer + endosperm s.s. The three main steps of the grain dissection allowing the collection of the three main compartments of interest (B-D).

For each heat shock treatment, five tagged spikes were harvested every day from anthesis to 350°Cd and then every two or three days until grain maturity. The spikes were sampled around 5:30 GMT, in order to minimize diurnal fluctuations in assimilate distribution. On each spike, within the central spikelet labelled for the date for anthesis, only the basal grains, grains G1 and G2 were sampled; their date of anthesis could be noted with more accuracy than from other florets. The date of all subsequent operations was related to the anthesis date specific to each grain. Grains were immediately weighed using a MX5 microbalance to give the fresh mass (Mettler-Toledo SAS, Viroflay, France) and grain volume was determined using a water displacement method (pycnometry). Dorsal and lateral images of grains were acquired under a stereomicroscope (MZ16F, Leica Microsystems, Wetzlar, Germany; magnification ×10 to ×40 depending on the developmental stage) to later measure the length/width and thickness, respectively, from images. G1 grains were oven-dried at 65°C for 48 h, then weighed to give the dry mass and calculate the water content. After weighing and imaging as described for G1, G2 were immediately dissected using tweezers and separated into OLs, endosperm and embryo (Fig 1). OLs were composed from colorless outer pericarp plus inner green pericarp [43, 54]; the endosperm compartment was composed from testa plus aleurone layer plus endosperm (*s*.*s*.). Material from each compartment was weighed to give the fresh mass then OLs were oven-dried at 65°C for 48h then weighed to give their dry mass and calculate their water content. After weighing, G2 endosperms were photographed under the stereomicroscope in the dorsal position for later measurement of their length and width from images. Then they were fixed for 2 h at room temperature in a 1:3 (v/v) acetic acid-alcohol mixture for later cell counting. As shown by Calderini *et al*. (1999) [18] and our previous experiments on the same wheat cultivar grown under the same conditions, G1 and G2 grains do not differ significantly in final dry mass and volume and are thus considered as equivalent. Therefore, endosperm dry mass was estimated as the difference between the dry mass of G1 grains and the sum of OLs and embryo dry masses of G2 grains.

### Endosperm cell counting

Endosperm cell number was determined by the method of Rijven and Wardlaw (1966) [56], as modified by Singh and Jenner (1982) [57]. In short, G2 endosperm nuclei were stained by the Feulgen reaction. For grains sampled up to 80°Cd after anthesis, endosperm cell nuclei were stained and dispersed on a glass slide, then immediately counted under the stereomicroscope. For grains older than 80°C, endosperm was treated for 24 h at 45°C in a 2% pectinase:cellulase (w/v) enzymatic solution and cell suspensions were obtained by vigorous stirring. When the stained nuclei density was not too high, the cell suspensions were dispersed on a glass slide and all nuclei were directly counted under the stereomicroscope. If there were too many stained nuclei to count directly, 10 samples of 10 μL per homogenized endosperm suspension were placed on 0.22-μm pore diameter nitrocellulose membrane filters (Millipore, Billerica, MA, USA). Each membrane was then photographed at ×10 magnification. Cells were then counted automatically using a cell counting macro of the image processing and analysis software ImageJ 1.53 (National Institute of Mental Health, Bethesda, Maryland, USA). For each endosperm, the mean cell number counted per spot is then brought to the final volume of homogenized endosperm suspension to estimate the total endosperm cell number.

### Analysis of trait dynamics over time and final value

The SAS (SAS Institute, 2002) General Linear Models (GLM) procedure was used to determine the statistical significance of differences in the final masses or dimensions of whole grains or compartments between the four temperature treatments. Means were compared using a Student-Newman-Keuls (SNK) test at the 5% level of significance. The growth curves, expressed as a function of thermal time, of whole grain or compartment fresh mass, volume and width and the growth curve of endosperm cell number were fitted to an asymmetrical 4-parameter Gompertz function equation [40]. The accumulation of dry mass in the whole grain or compartments was described by fitting a 3-parameter logistic function equation [40, 53]. Growth in length and water accumulation by the whole grain was described with a linear segmented model [58]. The growth function describing the observations most adequately (S2 Table; [53, 58, 59]) was chosen based on the homogeneity and values of residuals, biological coherence (e.g., shape, starting values and duration) and the Akaike’s Information criterion (AIC). Then, for each of the different grain or compartment variables, the non-linear fitting equations were compared simultaneously between treatments by parallel curve analysis [60] using the SAS Nlin procedure. For each grain trait, the most likely sets of parameters for each treatment were estimated and the comparison between parameters was based on AIC [61]. The durations of growth/accumulation were estimated directly from growth parameters of the Gompertz and logistic equations. For the traits considered (fresh mass, dry mass, volume and width), the rates of growth/accumulation were estimated by derivation of the Gompertz and logistic function equations; the maximal rate was estimated as the point when derivatives become null and the average rate was estimated over the duration between anthesis and maturity.

## Results

### Target heat shocks within the grains were consistent with the required temperature

Four heat shocks treatments were applied on plants for four consecutive days during the grain lag-phase (5 DAA) and/or during the grain filling phase (23 DAA) (S2A Fig). Apart from the days when heat shocks were applied, the target temperature within the grains was consistent (18.9°C) with the required air temperature (18.5°C).

During the first period of heat shock application (HS1), grain temperature increased up to 36.7°C on the 4^th^ day of heat shock application (S2B Fig) and the mean daily grain temperature was 23.6 and 23.3 °C respectively for HS1 and HS12 temperature treatments, compared with the required mean daily air temperature (23.8°C) (S3 Table). The control mean grain temperature was higher (but not significantly) than the applied air temperature: 19.6 ± 1.1 °C for both Control and HS2 treatments. The first heat shock (HS1) was applied long enough after anthesis to avoid both grain abortion and reduction of number of grains per spike. None of the heat shock treatments had a significant effect (P = 0.99) on the number of grains per spike which was on average 40.4 ± 2.8 grain spike^-1^.

During the second period of heat shock application, grain temperature increased up to 39.2°C on the 1^st^ day of heat shock application (S2C Fig) and the mean daily grain temperature was 25.2 and 24.8°C respectively for HS1 and HS12 temperature treatments (S3 Table). The control mean grain temperature was consistent with the applied air temperature: 19.1 ± 0.7 °C for both Control and HS1 treatments. Regardless of the small differences recorded between air and grain temperatures, overall, the grains have been well subjected to heat shocks at the required grain development periods. Later on, results are expressed according to grain temperature.

### Heat shocks modified the kinetics of whole grain growth

The temperature treatments, and in particular HS2 and HS12, slightly (not necessarily significantly) decreased the final values of most of the whole grain traits. Only the final values of whole grain dry mass, associated with grain width, were significantly (P < 0.0001) decreased by 15.3% and 12.4% for HS2 and HS12 treatments respectively compared with the Control (Table 1).

**Table 1:** Final masses and dimensions of basal mature grains from the central spikelets of wheat plants exposed to heat shocks.

Treatment	Fresh mass(mg grain^-1^)	Dry mass (mg grain^-1^)	Water content(% of fresh mass)	Volume (mm^3^ grain^-1^)	Length (mm grain^-1^)	Width (mm grain^-1^)	Thickness (mm grain^-1^)
**Control**	45.2 ± 5.6 **a**	42.6 ± 4.7 **a**	11.8 ± 0.7 **a**	57.6 ± 7.1 **a**	6.1 ± 0.2 **a**	3.4 ± 0.2 **a**	2.9 ± 0.3 **a**
**HS1**	43.7 ± 8.9 **a**	40.8 ± 7.3 **a**	12.3 ± 1.2 **a**	54.6 ± 7.4 **a**	5.9 ± 0.4 **a**	3.4 ± 0.2 **a**	3.0 ± 0.2 **a**
**HS2**	40.2 ± 5.9 **a**	36.1 ± 4.6 **b**	11.7 ± 0.6 **a**	51.3 ± 5.1 **a**	6.1 ± 0.3 **a**	3.2 ± 0.1 **b**	2.8 ± 0.2 **a**
**HS12**	42.0 ± 3.4 **a**	37.3 ± 3.5 **b**	11.0 ± 0.6 **a**	52.0 ± 3.2 **a**	6.2 ± 0.3 **a**	3.2 ± 0.2 **b**	2.8 ± 0.2 **a**

Heat shocks were applied either during the early-phase (HS1), the grain filling phase (HS2) or during both lag- and filling- phases (HS12) of grain development. Data are means ± 1 SD (*n* = 5 to 10). Within each column, means followed by different letters are significantly different at the 5% level (SNK test).

**Fig 2:**
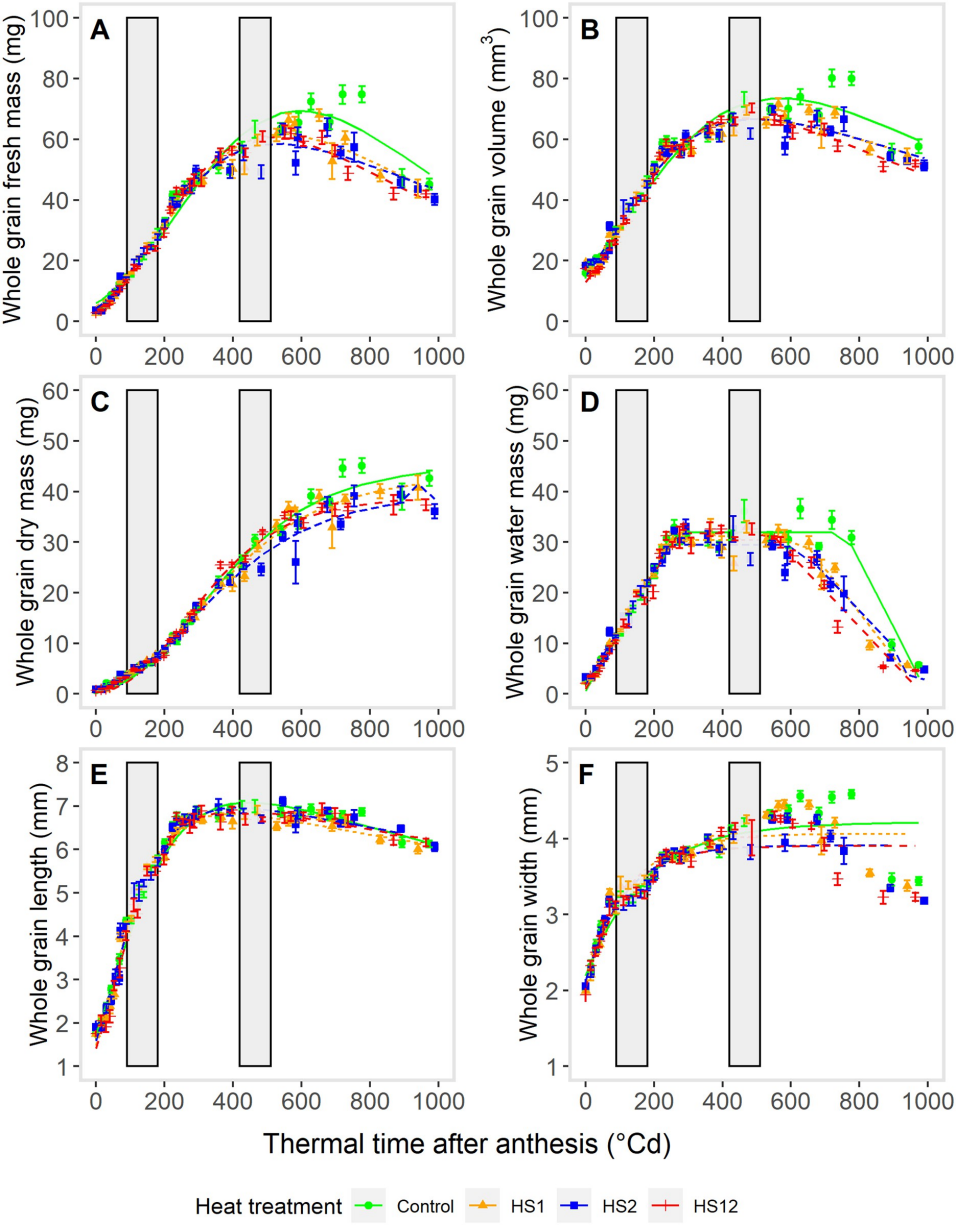
Effects of heat shocks on whole grain dimensions. Whole grain fresh mass (A), volume (B), dry mass (C), water mass (D), length (E) and width (F) during grain development after anthesis. Heat shocks (HS) were applied during the lag-phase (HS1), during the grain filling-phase (HS2) or during both the lag-phase and filling-phase (HS12) of the grain development, compared to Control. Each point corresponds to the mean of measurements on 10 basal grains from central spikelets sampled on 5 spikes. Lines represent the growth functions fitted to the observed values. Growth functions were selected on the base of the growth curves obtained for all treatments pooled (S2 Table). Vertical grey bars indicate the times and duration of exposure to heat shocks (~90°Cd and ~420°Cd after anthesis for respectively HS1 and HS2 during 4 days (~95°Cd).

**Fig 3:**
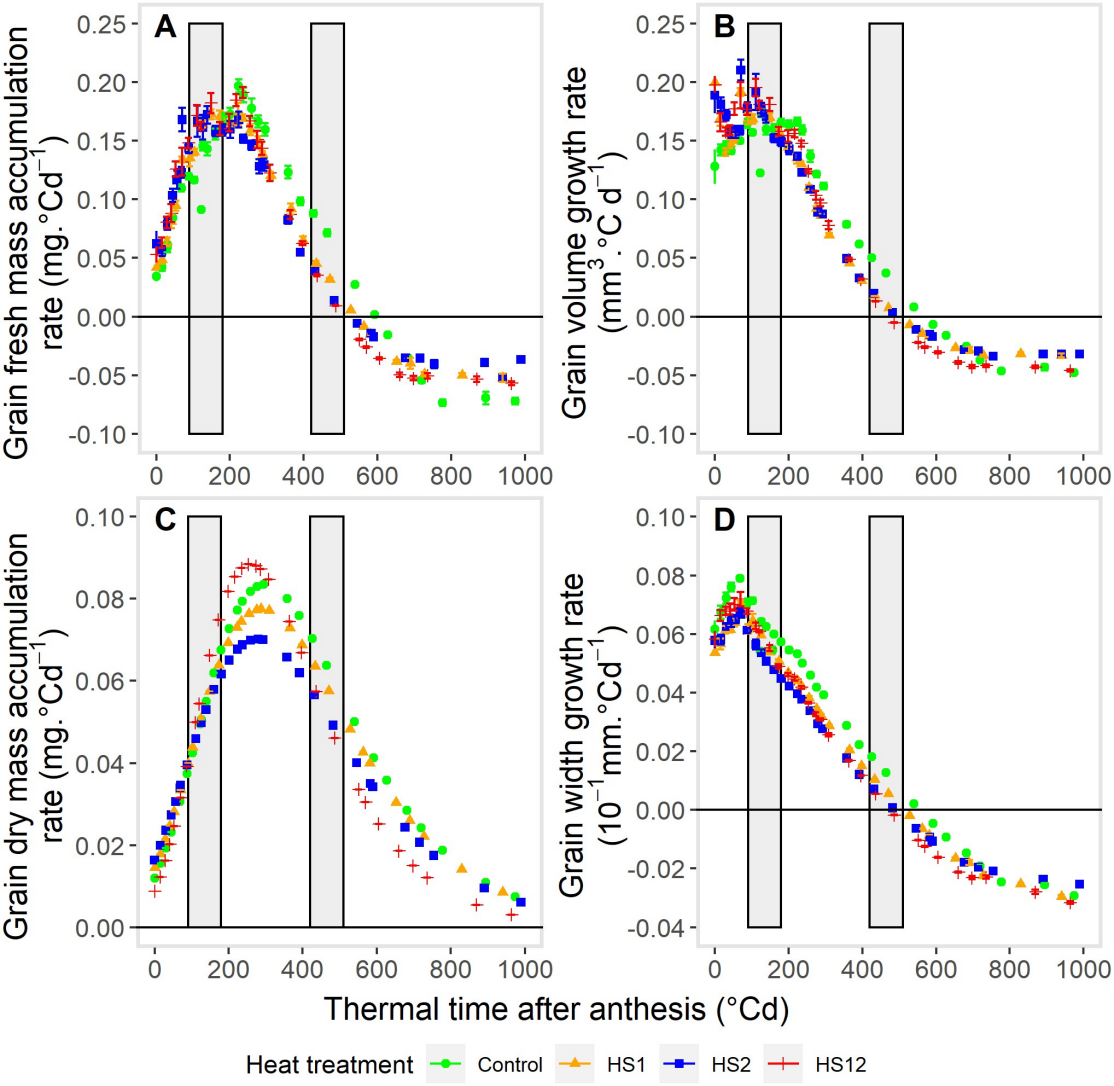
Effects of heat shocks on whole grain growth rates. Whole grain fresh mass accumulation rate (A), volume growth rate (B), dry mass accumulation rate (C), and width growth rate (D) during grain development after anthesis. Heat shocks (HS) were applied during the lag-phase (HS1), during the grain filling-phase (HS2) or during both the lag-phase and filling-phase (HS12) of the grain development, compared to Control. Each point corresponds to the values estimated from the derivative of the fitted growth functions (S2 Table). Vertical grey bars indicate the times and duration of exposure to heat shocks (~90°Cd and ~420°Cd after anthesis for respectively HS1 and HS2 during 4 days (~95°Cd).

For each trait, the growth curves had a similar pattern for the different temperature treatments (Fig 2). Regardless of the trait, compared with the Control, heat shock treatments modified the growth curves; this is particularly noticeable after the 2^nd^ heat shock treatment. For each trait, fitting the growth functions (Fig 2) allowed a more reliable comparison between temperature treatments by the estimation of the maximal value of each trait in each treatment (Table 2), as well as the growth rate (Table 3) and the duration of the growth processes (Table 4). For all treatments, grain fresh mass and volume increased up to a maximum before decreasing before the end of the grain-filling period (Fig 2A and 2B). Their maximum estimated values were decreased by heat shock treatments compared to the control (P>F < 0.0001), e.g., by 15.6 and 9.3% for grain fresh mass and volume respectively between control and HS2 treatment (Table 2). Maximum values were reached more rapidly for HS12 treatment (Table 3, Fig 3A and 3B) and earlier (Table 4, Fig 3A and 3B) for allheat shock treatments.

**Table 2:** Estimated maximum values of the various traits of basal grains from the central spikelets of wheat plants exposed to heat shocks.

Treatment	Fresh mass (mg grain^-1^)	Dry mass (mg grain^-1^)	Water mass (mg grain^-1^)	Volume (mm^3^ grain^-1^)	Length (mm grain^-1^)	Width (mm grain^-1^)	Thickness (mm grain^-1^)
**Control**	69.37 ± 0.82	45.3 ± 1.1	32.0± 0.5	73.5 ± 0.7	6.84 ± 0.05	4.45 ± 0.03	3.44 ± 0.04
**HS1**	61.72 ± 0.77	43.3 ± 1.2	30.4 ± 0.5	66.7 ± 0.5	6.66 ± 0.03	4.27 ± 0.03	3.27 ± 0.03
**HS2**	58.52 ± 0.78	39.8 ± 1.1	29.7 ± 0.5	66.7 ± 0.5	6.79 ± 0.04	4.13 ± 0.03	3.26 ± 0.03
**HS12**	60.90 ± 0.71	39.0 ± 0.9	31.7 ± 0.5	67.2 ± 0.7	6.74 ± 0.04	4.16 ± 0.03	3.25 ± 0.03

Heat shocks were applied either during the lag-phase (HS1), the grain filling-phase (HS2) or during both phases (HS12). For each grain trait, the non-linear fitting equations were compared simultaneously between treatments (Ross, 1984) and the more likely model was chosen based on the AIC criterion. Maximum values were estimated from the parameters of the most likely model. Data are estimated maximum value ± approximated SE.

**Table 3:** Estimated rate of growth of basal grains from the central spikelets of wheat plants exposed to heat shocks.

Treatment	Fresh mass (mg °Cd^-1^)	Dry mass		Water mass		Volume (mm^3^ °Cd^-1^)	Length (mm °Cd^-1^)	Width (mm °Cd^-1^)
		(mg °Cd^-1^)	(mg day^-1^)	Accumulation (mg °Cd^-1^)	Dehydration (mg °Cd^-1^)			
**Control**	0.116	0.057	1.67	0.12	-0.13	0.130	0.020	0.00360
**HS1**	0.114	0.052	1.57	0.12	-0.01	0.134	0.024	0.00404
**HS2**	0.110	0.048	1.46	0.11	-0.07	0.134	0.022	0.00387
**HS12**	0.120	0.060	1.74	0.12	-0.07	0.142	0.022	0.00395

Heat shocks were applied either during the lag-phase (HS1), the grain filling-phase (HS2) or during both phases (HS12). Rates were estimated from the parameters of the nonlinear functions fitted to the growth curves of changes in grain mass, volume and dimensions. Except for length and water mass, rates of accumulation or growth refer to the average rate estimated between anthesis and maturity. Rate of growth in water mass was estimated as the slope of (i) the first linear part (accumulation) or (ii) the third linear part (dehydration) of the linear segmented function adjusted to water mass values. Rate of growth in length was estimated as the slope of the first linear part of the linear segmented function adjusted to length values.

**Table 4:** Estimated duration of growth of basal grains from the central spikelets of wheat plants exposed to heat shocks.

Treatment	Fresh mass (°Cd)	Dry mass		Water mass		Volume (°Cd)	Length (°Cd)	Width (°Cd)
		(°Cd)	(Days)	Accumulation (°Cd)	Dehydration (°Cd)			
**Control**	595.8 ± 8.1	711.5 ± 24.2	38.5 ± 1.3	240.0 ± 14.1	764.8 ± 16.7	567.7 ± 9.5	238.8 ± 5.0	586.2 ± 10.1
**HS1**	541.6 ± 8.9	700.2 ± 25.6	37.1 ± 1.4	221.8 ± 15.9	660.9 ± 21.1	497.1 ± 7.4	203.3 ± 3.8	512.1 ± 10.6
**HS2**	528.0 ± 10.1	708.7 ± 28.6	37.5 ± 1.5	210.0 ± 17.7	550.8 ± 26.3	497.1 ± 7.4	216.0 ± 4.3	488.8 ± 11.7
**HS12**	505.5 ± 8.0	614.3 ± 22.3	31.2 ± 1.1	225.3 ± 19.3	544.1 ± 14.3	471.7 ± 8.4	215.0 **±** 4.0	473.4 ± 10.2

Heat shocks were applied either during the lag-phase (HS1), the grain filling-phase (HS2) or during both phases (HS12). For each grain trait, the non-linear fitting equations were compared simultaneously between treatments (Ross, 1984) and the most likely model was chosen based on the AIC criterion. Durations were estimated from the parameters of the most likely model. For grain fresh or water masses, volume, length or width, the duration of accumulation or growth was calculated as the time from anthesis at which the maximum value was attained (*i*.*e*., when growth rates were null). For grain water mass, an additional duration was calculated as the time between grain anthesis and the beginning of grain dehydration. For dry mass, the duration of accumulation was calculated as the time (in °Cd or days) when 95% of the final grain dry mass was attained. Data are estimated duration ± approximated SE.

For all temperature treatments, grain dry mass followed a classical sigmoid pattern (Fig 2C). Maximal estimated grain dry mass was affected by all heat shock treatments (P>F <0.0001) and in particular, by HS2 and HS12 treatments (with a decrease of 12 and -14% for HS2 and HS12 treatments compared with the Control (Table 2)). The dynamics of grain dry mass accumulation rate were however different (not necessarily significantly; Fig 3C). Except for HS12 treatment, the average rate of grain dry mass accumulation tended slightly or significantly (for HS2) to decrease in presence of heat shock compared with control (Table 3) while the duration was not affected by HS1 or HS2 (Table 4). Note that for some unexplained reason (probably a lesser quality of fitting due to some weird points around 360–400°CDAA), a statistical significant decrease (P>F < 0.01) of both the maximal and average rates of dry matter accumulation in HS2 compared to Control occurred whereas HS2 treatment was applied after the moment in which the maximum dry mass accumulation rate was reached. In contrast, HS12 slightly increased the rate of grain dry mass accumulation but significantly reduced the duration of accumulation (by 13.7% and 19% respectively when expressed in °Cd and days) compared to the Control.

The level of the water mass plateau significantly decreased when affected by HS1 or HS2 (by -5 and 7% respectively for HS1 and HS2 compared to the Control; Fig 2D, Table 2). While the average rate of grain water accumulation was not affected by heat shock treatments, grains under heat shock treatments dehydrated less rapidly compared to the Control (Fig 2C). In the same way, the duration of grain water accumulation was not affected by heat shock treatments, but the duration of water plateau was decreased by heat shock and especially by HS12 treatment (by 13.6, 28.0 and 28.9% respectively for HS1, HS2, HS12; Table 4, Fig 2D). Note that heat shock treatments affected the grain water and dry mass to a similar extent, as demonstrated by the absence of response of grain water content (Table 1).

As expected, grain length was mainly decreased by HS1 treatment (Table 2); due to shorter duration of length growth (Table 4) not fully compensated by an increase of the average grain length growth rate (Table 3). The dynamic of grain width growth followed similar pattern to fresh mass accumulation or volume growth (Fig 2E). The value of the final grain width could not be properly estimated but the maximum value of grain width was estimated with confidence and was attained more rapidly (Table 3; Fig 2D) and earlier (Table 4; Fig 2D) when plants were exposed to heat shocks.

Overall, heat shock treatments tended to decrease maximum estimated values of traits by decreasing the durations of the various accumulation/growth processes that were partly compensated by increased rates of growth. Moreover, it is also worthwhile that for most of the grain growth processes, HS12 treatment was not more detrimental to grain growth than the HS1 or HS2 treatments.

### Heat shocks reduced the maximum number of endosperm cells but did not affect OLs dry mass

**Fig 4:**
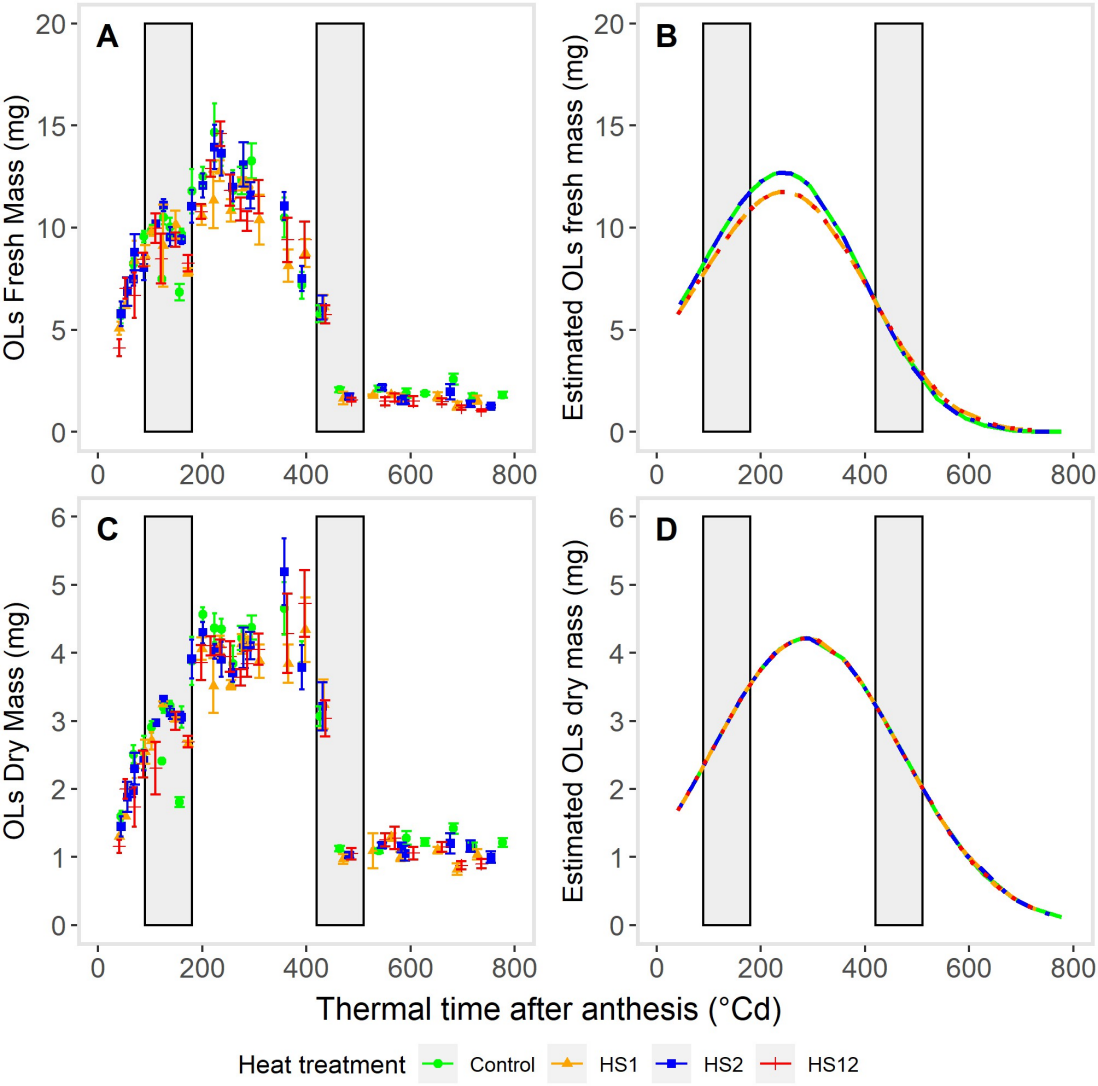
Effects of heat shocks on OLs growth. OLs fresh mass accumulation (A,B), dry mass accumulation (C,D) during grain development after anthesis. Each point on A or C graphs corresponds to the mean of measurements on 10 basal grains from central spikelets sampled on 5 spikes. Curves on graphs B and D represent the fitted growth functions (Gompertz with maxima) to the observed values of OLs fresh mass (B) or dry mass (D). Heat shocks (HS) were applied during the lag-phase (HS1), during the grain filling-phase (HS2) or during both the lag-phase and filling-phase (HS12) of the grain development, compared to Control. Vertical grey bars indicate the times and duration of exposure toheat shocks.

Dissection of OLs (Fig 1) allowed us to follow their dynamics of fresh and dry masses under the different heat shock treatments (Fig 4). The fresh and dry masses of the pericarp showed similar dynamics from anthesis to grain maturity for the Control and for the three temperature treatments. Pericarp fresh mass linearly increased from anthesis to ~230°Cd, then rapidly decreased between ~250°Cd and ~450°Cd and from this time remained stabilized until maturity. Heat shock during the lag-phase (HS1) or heat shocks during both lag-and filling-phases (HS12) slightly decreased OLs fresh mass dynamics 4, by mainly reducing the average OLs growth rate (Fig 4A and 4B). The estimated maximum OLs fresh mass is reduced by 7.5% either by a single early heat shock (HS1) or by an early heat shock followed by a later heat shock (HS12) compared to Control (or HS2) (Fig 4B). Pericarp dry mass linearly increased up to a maximum attained at ~380°Cd, before decreasing rapidly until ~450–500°Cd and then remained stabilized between ~450°Cd and the physiological maturity. None of the heat shock treatments had any effect on the dynamics of OLs dry mass accumulation (Fig 4C and 4D; S4 Table), with a maximum value estimated to 4.20 mg, all temperature treatments pooled. Note that regardless of the temperature treatment, the fit of the growth function to OLS dry mass values did not well capture the maximum values around 450°Cd, due to a higher variability of OLs dry mass at that time.

**Fig 5:**
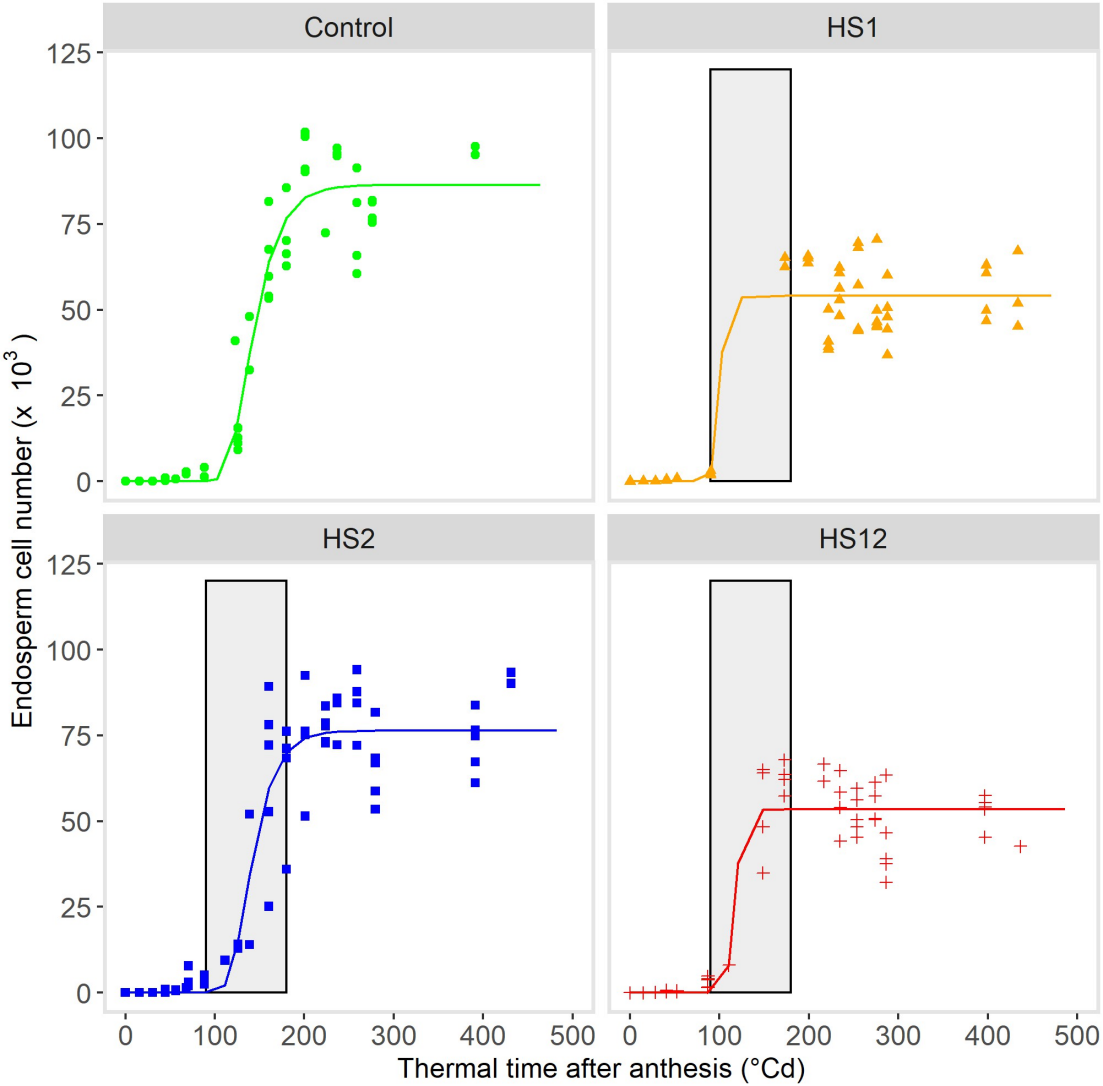
Effects on heat shocks on endosperm cell number. Heat shocks (HS) were applied during the lag-phase (HS1), during the grain filling-phase (HS2) or during both the lag-phase and filling-phase (HS12) of the grain development, compared to Control. Each point corresponds to cell counting in one grain (n = 5 per each sampling date). Curves correspond to the growth function (Gompertz with maxima) fitted to the observed values over time after anthesis. Vertical grey bars indicate the times and duration of exposure to heat shocks (~90°Cd and ~420°Cd after anthesis for respectively HS1 and HS2 during 4 days (~95°Cd).

Endosperm cell multiplication dynamics between anthesis and 450°Cd are reported on Fig 5. The average maximum number of endosperm cells was 86 373, 54 034, 76 399 and 53 537 for control, HS1, HS2, HS12, respectively (Fig 5). Another endosperm cell count from grains aged approximately 700°Cd confirmed that there was either no further cell proliferation or a slightly decrease after 450°Cd (72818, 50340, 72748, 50805 for Control, HS1, HS2, HS12, respectively). Clearly, heat shock during the lag-phase of grain development significantly reduced the maximum number of endosperm cells by 37% and 38% in HS1 and HS12 respectively (P<0.0019). The duration and rate of cell proliferation were estimated by combining the data for Control and HS2 on one hand, and for HS1 and HS12 on the other hand, as a way of increasing the number of replicates and the power of statistical tests. The average rate of cell proliferation during the linear phase of cell proliferation (*i*.*e*., 100 and 200°Cd after anthesis) was 512 cells °Cd^-1^ for HS1-HS12 treatments, significantly lower (P< 0.0016) than 953 cells °Cd^-1^ for Control-HS2 treatments. Moreover, the duration of the cell proliferation period was shorter (P<0.0001) for HS1-HS12 treatments at 186 ± 20°Cd than for Control-HS2 treatments at 220 ± 10 °Cd. Therefore, the reduction in endosperm cell number was the result of a reduction in both the duration and rate of cell proliferation.

### The effect of early heat shocks (HS1 or HS12) on the growth of the whole grain or its compartments was pronounced at the end of the lag-phase

The estimated duration of endosperm cell proliferation was used to assess the effect of HS1 and HS12 treatments on the dimensions of the whole grain (Table 5) and of its compartments (Table 6) at the end of the lag-phase, considering that the end of the phase of endosperm cell proliferation marked the end of the lag-phase. Except for the dry mass of OLs, all the whole grain (Table 5) and compartments (Table 6) were significantly reduced by heat shocks during the lag-phase (HS1 and HS12). For example, the dry mass of the whole grain and endosperm were 18% and 21% lower, respectively, for HS1 than for Control. The volume of the whole grain and the endosperm were reduced by 16% and 21% respectively, in HS1 compared to Control. At the end of the lag-phase, there was no difference (at the 5% level) between HS1 and HS12 in any of the whole grain or compartment traits. As expected, at the end of the lag-phase, HS2 resulted in no change in fresh or dry mass of either whole grain or compartments compared to Control. Thus, at the end of the lag-phase, the size of the grain, in addition to the number of endosperm cells, was already reduced by an early heat shock, but at maturity the effect of a single early heat shock was no longer observed (Table 1).

**Table 5:** Masses and dimensions of basal grains from the central spikelets at the end of the lag-phase.

Treatment	Fresh mass (mg grain^-1^)	Dry mass (mg grain^-1^)	Water content (% of fresh mass)	Volume (mm^3^.grain^-1^)	Length (mm grain^-1^)	Width (mm grain^-1^)	Thickness (mm grain^-1^)
**Control**	41.4 ± 5.0 **a**	10.6 ± 2.8 **a**	74.6 ± 4.0 **a**	59.5 ± 4.8 **a**	6.7 ± 0.4 **a**	3.8 ± 0.1 **a**	3.0 ± 0.2 **a**
**HS1**	31.5 ± 1.5 **b**	8.7 ± 0.4 **b**	72.9 ± 0.6 **a**	50.1 ± 1.8 **b**	5.8 ± 0.2 **b**	3.6 ± 0.1 **ab**	2.8 ± 0.2 **b**
**HS2**	38.6 ± 2.9 **a**	11.3 ± 0.7 **a**	71.5 ± 0.6 **a**	55.5 ± 3.4 **a**	6.6 ± 0.2 **a**	3.8 ± 0.2 **a**	3.0 ± 0.2 **a**
**HS12**	29.1 ± 4.1 **b**	7.6 ± 1.3 **b**	72.5 ± 0.8 **a**	47.1 ± 4.9 **b**	5.8 ± 0.4 **b**	3.5 ± 0.1 **b**	2.8 ± 0.2 **b**

Wheat plants were exposed to heat shock either during the lag-phase (HS1), the grain filling-phase (HS2) or during both lag- and filling- phases (HS12) of grain development. Data are means ± 1 SD (*n* = 5 to 10). Within each column, means followed by different letters are significantly different at the 5% level (SNK test).

**Table 6:** Fresh and dry masses and volumes of the endosperm and OLs at the end of the lag-phase.

Treatment	Endosperm			OLs	
	Fresh mass (mg grain^-1^)	Dry mass (mg grain^-1^)	Volume (mm^3^ grain^-1^)	Fresh mass (mg grain^-1^)	Dry mass (mg grain^-1^)
**Control**	22.3 ± 3.8 **a**	8.5 ± 2.6 **a**	36.9 ± 3.3 **a**	14.7 ± 3.2 **a**	4.4 ± 0.5 **a**
**HS1**	14.4 ± 1.9 **c**	6.7 ± 0.8 **b**	29.3 ± 1.9 **b**	10.6 ± 1.1 **b**	4.1 ± 0.4 **a**
**HS2**	19.1 ± 1.4 **b**	9.3 ± 0.9 **a**	37.1 ± 2.3 **a**	13.9 ± 2.4 **a**	4.0 ± 0.2 **a**
**HS12**	12.9 ± 0.9 **c**	5.7 ± 1.4 **b**	25.4 ± 4.8 **b**	10.8 ± 0.8 **b**	3.9 ± 0.6 **a**

Wheat plants exposed to heat shocks during the lag-phase (HS1), the filling-phase (HS2) or both phases (HS12). For OLs, it was not possible to measure the volume as grains were dissected. The end of the lag-phase was estimated as the end of endosperm cell multiplication (*i*.*e*., 220°Cd for C and HS2 treatments and 186°Cd for HS1 and HS12 treatments). Data are means ± 1SD (n = 5). Within each column for a given developmental stage, means followed by different letters were different at a 5% level of significance (SNK test).

## Discussion

Post-anthesis stress due to moderately or acute high temperatures causes an array of morpho-anatomical, physiological, biochemical, and molecular changes at whole plant, organ, tissue, cell and molecular levels [25, 35, 62]. In cereals, under most heat conditions, the reduction in grain growth does not appear to be due to a lack of assimilates [21, 51, 63] or to a reduced translocation of assimilates from vegetative tissues to grains [64, 65]. This suggests that part of the response to elevated temperature results from direct and autonomous effects on the grain. Here we report the effects of post-anthesis heat shocks (HS) on dynamic patterns of grain traits, namely grain and OLs fresh and dry mass accumulation, grain volume expansion and water accumulation and endosperm cell proliferation (Figs 1–5). The patterns are similar to those previously found in studies on wheat [39, 40, 43, 51, 66] or on other cereals such as maize or sorghum [67, 68]. In particular, our data allowed the study of the effect of heat shocks applied during the lag-phase of grain development, on grain traits at the end of this lag-phase and consequently at grain maturity.

### Grain traits at the end of the early-phase of grain development were strongly reduced by a single early heat shock

Compared to Control treatment, both HS1 and HS12 treatments reduced all the whole grain traits (except the grain water content expressed in % of grain fresh mass) at the end of the lag-phase of grain development (Table 5). Moreover, HS1 or HS12 reduced the maximum endosperm cell number. This was the result of both a reduction of rate and duration of cell multiplication. Similar effects of high temperatures applied as heat shock were previously reported for the maximum number of cells in endosperm of maize [69] or in rice [70]. However, these results are inconsistent with literature on wheat [26, 51] where high temperatures were found to have no effect on maximum endosperm cell number due to a higher rate that fully compensated a shorter duration of cell multiplication. Apart from genotypic difference, the discrepancy between the literature and the present study may be related to the timing, duration and in particular to the intensity of the heat stress: in the latter [26, 51], applied temperatures were moderately high (less than 27°C in both studies) even when applied as soon as 3 days after anthesis [51]. However, contrary to moderately high temperatures, heat shocks (38 to 42°C for 30 min) have been shown to have major effects on the processes of cell division [71]. Stone and Nicolas (1995) [30] suggested that there are specific effects of very high temperatures (40°C for 6 hours a day during 5 days), which are induced above some temperature threshold and that are different from those of moderately high temperature.

In contrast, slightly or no effect of HS1 or HS12 was observed on OLs fresh and dry mass at the end of the lag-phase. Unfortunately, in our experimental conditions, we were unable to measure the volume bounded by the OLs as their dissection for weighing was a destructive method. The use of imaging non-destructive methods [72] to measure the internal volume available for endosperm growth would help to better understand the effect of high temperatures on the role of OLs on final grain weight. The absence of effect of HS1 or HS2 on OLs mass is in contradiction with results recently obtained by Herrera and Calderini (2020) [43]. These authors reported a negative impact of high temperature (+6°C above the ambient post-anthesis temperature in field conditions) on maximum pericarp fresh and dry masses and water content; the maximum pericarp values are suggested to be closely related to the final grain weight. First, the discrepancy between the literature [43] and the present study may be related to genotypic differences, as genetic variability of response of grain development to high temperature has been reported [28, 73]. In addition, in our study, HS1 or HS12 were applied at ~5 DAA for 4 consecutive days when there is no more cell multiplication in the pericarp and only still pericarp cell enlargement [41, 74]. Even if, in the present study, the intensity of temperature was as high as 38°C, HS1 and HS12 treatments were probably not long enough to significantly impair the pericarp growth through cell enlargement. In contrast, in Herrera and Calderini (2020) [43], higher temperatures were applied on a longer duration (from anthesis to 16 DAA) and during night, as night temperature has been shown to affect more negatively cell enlargement than similar day temperature [75–77].

Then, in the present study, at the end of the lag-phase, the reduction of the grain fresh and dry mass and volumes was mainly related to the reduction of endosperm mass and volume and in particular in maximum cell number and not to the OLs fresh and dry mass. In cereal grains, the maximum number of endosperm cell has been related to grain yield, as it has been shown to represent a potential number of sites for starch deposition and then defining a potential sink or storage capacity [47, 57, 78], assuming no limitation in assimilate supply within the endosperm. Then, HS1 and HS12 should have led to a significant reduction of grain dry mass at maturity.

### But final grain dry mass was not negatively affected by a single early heat shock

The absence of effect of HS1 on final grain dry mass compared to Control treatment is related to an increase of the duration of the grain-filling phase of grain development (Table 7), suggesting that a single early heat shock (HS1) did not impair the subsequent capacity of the grain to accumulate assimilate and to synthesise starch during the filling-phase. From the maximal volume of endosperm and the maximum cell number in the endosperm, it was possible to estimate the mean maximum volume of endosperm cells assuming that the volume of the endosperm was completely occupied by cells with no space between cells (this hypothesis was supported by the observation under microscope of transversal sections of growing wheat grains [54]). Endosperm cell volume was estimated to be 0.77 × 10^−3^, 1.01 × 10^−3^ mm^3^ for Control and HS1, respectively (there were 0.78 × 10^−3^, 1.03 × 10^−3^ mm^3^ for HS2 and HS12, respectively). Therefore, heat shock during the lag-phase increased subsequent endosperm cell volume (by ~31%) after the heat shock release and appeared to compensate for the reduction in the maximum number of endosperm cells. This phenomenon of compensation, *i*.*e*., a reduction in cell number accompanied by an increase in cell size leading to a maintenance of the organ size even when cell proliferation is disrupted by stresses, has been reviewed several times [79–81]. This compensatory mechanism occurs only in determinate organs [81] such as leaves, fruits or grains and has been reported for example in fruit tomato [82], cucumber [83] or rice grains in transgenic plants and mutants defective in regulators of cell proliferation [84]; moreover, it has been suggested in wheat grain in response to shading treatments [85]. Under heat stress, cell expansion, permitted by cell wall remodelling [86] and in this study probably not prevented by restricted OLs enlargement, might drive sustained grain filling after the first heat shock and recovery of the normal temperature. So, contrary to what might be expected [47], if this putative compensatory mechanism occurs, the maximum cell number is not always correlated to final grain dry mass under heat treatment. In the present study, even though the number of values (corresponding to the 4 treatments) was low, our results showed no significant correlation between the maximum endosperm cell number and the final grain dry mass or the final endosperm dry mass (respectively R^2^ = 0.06 and R^2^ = 0.03; P = 0.76 and P = 0.81; S3A and S3B Fig). Note that a weak positive relationship has been found between the final grain dry mass and the maximum water mass (R^2^ = 0.31; P = 0.44; S4 Fig). Although not statistically significant, due to the small range of maximum grain water mass variations among treatments, this result is in accordance with the literature [45, 74], that proposed water accumulation within the grain as a major driver of grain growth and filling and of synthesis of storage products.

**Table 7:** Duration, expressed in °Cd, of the different phases of the grain development for each heat shock treatment.

	Duration (°Cd)		
	Lag-phase	Filling-phase	Whole grain development
**Treatment**			
**Control**	215	497	711
**HS1**	183	517	700
**HS2**	227	482	709
**HS12**	188	426	614

The duration was calculated as the difference between end time and the beginning time of each phase. The end of the lag-phase (or the beginning of the filling-phase) was estimated as the time when cell multiplication in the endosperm ends. The end of the filling-phase was estimated by the time when 95% of the final dry mass of the grain is attained using a 3-parameters logistic growth curve.

### Under our experimental conditions, the lag-phase is less sensitive to heat shock than the grain filling-phase

The putative compensatory mechanism described above might explain why, in the present study, the lag-phase was not found the most sensitive grain developmental phase compared to the grain filling-phase, in contradiction with the literature [35, 57, 78]. Indeed, at maturity, whole grain dry mass and width (Table 1) as well as maximum values of all the whole grain traits (Table 2) were reduced by a single heat shock occurring during the filling-phase (HS2) compared to the HS1 treatment (or Control treatment). This was related to a decrease of both the duration and rate of the filling-phase compared to the Control treatment (Table 7). This result is in agreement with many studies reporting a major impact of high temperatures (chronic or acute) occurring during the filling-phase on grain dry mass accumulation [21, 30, 51, 87] and is consistent with transcriptome analysis where heat treatments compressed the expression of genes that regulate metabolic processes of grain development [88, 89]. As expected, the endosperm cell number was not affected by HS2 treatment compared to the Control; but the estimated cell volume was not reduced either despite a reduction in final grain dry mass. This result could either be due to a compensation between endosperm cell number and cell volume that we were unable to identify or to the fact that HS2 was applied too late in relation to the maximum endosperm cell expansion, then limiting the impact of HS2 on endosperm cell volume. In the latter case, the effect of HS2 would rather be due to a reduction in dry matter accumulation within the grain related to a decrease of starch synthesis. In particular, among the enzymes regulating starch synthesis, the soluble starch synthase (SSS) is the most sensitive one to heat stress [90–93]. All together, these results suggest independent effects of a late heat shock during the grain-filling phase on grain expansion and dry mass accumulation, and are consistent with the literature where processes related to dry biomass accumulation and expansion in the grain have different sensitivities to heat [40].

### The effects of heat shocks during both the lag- and filling-phases of grain development on final grain dry mass werenot additive

Various studies [19, 94] reported that the effects of brief exposures to high temperature during early grain development followed by sustained periods of moderately high temperatures were not additive. Our results are in agreement with these studies [19, 94] as under our experimental conditions, two consecutive heat shocks during both the lag- and the filling-phases (HS12) reduced the final grain dry mass as much as a single heat shock during the filling-phase (HS2) (Table 1; Fig 1C). This non-additivity might be explained by compensation phenomena between some of the underlying processes (cell multiplication, cell enlargement, starch synthesis…). Indeed, HS12 treatment decreased by ~37% the maximum endosperm cell number compared to the Control treatment. However, between the first and second application of heat shocks, grains in HS12 treatment were exposed to temperatures similar to the Control ones during 18 days (S2A Fig). This allowed a full or partial compensation of the reduced number by an increase of endosperm cell volume, as shown for HS1 treatment; endosperm cell volume was estimated at 1.03 × 10^−3^ mm^3^ for HS12, similar to endosperm cell volume estimated for HS1 treatment and larger than one of Control and HS2. The stimulated enlargement of endosperm cell would contribute to maintain a sink activity as far as the starch synthesis would not be affected by the second heat shock. The fact that the effects of HS12 and HS2 on final grain dry mass did not differ, despite different endosperm cell number and estimated cell volume, suggest that the reduction in final dry biomass was mainly due to the effect of the second heat shock during the grain-filling phase on grain dry mass accumulation per se, and in particular on starch synthesis (as suggested for HS2 treatment).

If this interpretation is relevant, it seems to be in contradiction with the one proposed by Stone *et al*. (1995) [19]. According to their hypothesis, the reduced impact of two successive heat shocks compared to a single heat shock results from the reduction in potential yield caused by the early first heat shock on the endosperm cell number or on grain volume. In other words, if the negative effect of a heat shock is “proportionate” to the grain size, in case of smaller grains resulting from a first early heat shock, the second heat shock would have a lesser impact than on bigger grains from control treatments. In our case, this interpretation proposed by Stone *et al*. (1995) [19] is not consistent with the observation of an increased cell expansion and dry matter accumulation rate (Table 3) after the first heat shock.

If the putative compensatory mechanism between endosperm cell number and estimated cell volume is confirmed, the underlying molecular mechanism is far from to be elucidated [81]. The production of heat shock proteins (HPSs; e.g. [95, 96]) could be one of the molecular pathways. Wheat HSPs are synthesised in all tissues including developing and mature grains [97, 98]. In the HS12 treatment, it is possible that HSPs produced during the first heat shock protect the enzymes involved either in cell expansion or grain filling [50, 99, 100].

### Challenges related to the role of the compensatory mechanism between cell number and size in response to heat shock

In this study, endosperm cell volume was estimated by the calculated ratio between the maximum endosperm volume to the maximum cell number. First, this estimation does not consider the spatial variability of the cell volume within the grain [101]; second, it would be more accurate to measure directly endosperm cell volume, for example using non-destructive 3D-imaging techniques (e.g. [42]).

In the present work, responses of grains to heat shocks were studied only on the basal grains taken from the central spikelets of the spikes. However, the effects of high temperature on final grain dry mass are different depending on grain position on the spike and within the spikelet [18, 94]: basal grains of the basal spikelets were more sensitive to high temperatures than those of the apical spikelets and distal grains within a spikelet were more sensitive to heat than proximal grains. Under control conditions, the differences in final grain mass, at least between grains within the same spikelet, have been attributed to limited assimilate availability during the grain filling-phase for distal grains compared to basal grains, thus implying that distal grains are more source limited than sink limited [66]. The existence of the putative compensatory mechanism described for the basal grains of central spikelets should be checked as a function of the position of the grains on the spike. For grains that are more source limited than sink limited, it is likely that the compensation between endosperm cell number and cell volume under heat conditions could not be as large as in the basal grains. This could support the previous results [18, 94] and explain the lower stability of distal final grain dry mass compared to that of basal grains in response to high temperatures.

In this study, the thermal treatments (intensity, timing and duration of heat shock) were selected to be as far as possible representative of natural conditions possibly encountered under field conditions. Moreover, the use of the C3-GEM platform allowed natural lighting and the experiments were conducted under non-limiting conditions, with the absence of biotic or abiotic stress such as evaporative demand (low-VPD conditions) and soil water deficit. However, during the grain filling period in a large number of field conditions, heat and drought are often concomitant [102, 103]. The ability of grains to compensate endosperm cell number by cell enlargement in response to a combination of heat and water stress should be checked.

This study reports responses of wheat grain to be different from the responses to moderately high temperature, the main difference being the absence of effect of an early heat shock on final grain dry mass. These results thus lend further support to the view that both the effects of chronic and acute high temperatures need to be further investigated [19, 104]. Moreover, the largest effect of an early heatshock during the lag-phase (HS1 and HS12) compared to the Control treatment have been observed on the maximum endosperm cell number and expansion. Even this study certainly presents some limits to be kept in mind, it reports in the wheat grain for the first time a possible compensation between endosperm cell number and size in response to early heat shock. Molecular studies have begun to provide some insight into the phenomenon of compensation cell number/size, at least in vegetative organs such as leaves [81]. Further studies are needed at the grain level to clarify which thermal conditions induce these mechanisms, and investigate their genetic and molecular basis. This would improve the understanding of post-anthesis wheat tolerance to heat shocks.

## Supporting information

S1 Fig Weather conditions from sowing to anthesis. Mean day temperature (°CDAA) and daily rainfall (mm) (A) and daily solar radiation (MJ m^-2^) (B).
(TIF)

S2 Fig Grain temperature throughout the experiment. Daily mean grain temperature (A) over time from anthesis to grain maturity and hourly mean grain temperature over the four days of exposure during the first heat shock (lag-phase) (B) or during the second heat shock (filling-phase) (C).
(TIF)

S3 Fig Relationship between the maximum endosperm cells and the final grain dry mass (A) and endosperm dry mass (B). For each heat shock treatment, each point corresponds to the estimated mean value by the fitting of growth functions to the observed values (3-parms logistic and Gompertz with maxima for dry mass and endosperm cell number, respectively (S1 Table)). Heat shocks (HS) were applied during the lag-phase (HS1), during the grain filling-phase (HS2) or during both the lag-phase and filling-phase (HS12) of the grain development, compared to Control.
(TIF)

S4 Fig Relationship between the maximum grain water mass and the final grain dry mass. For each temperature treatment, each point corresponds to the estimated mean value by the fitting of growth functions to the observed values (3-parms logistic and segmented linear function for grain dry mass and water mass, respectively (S1 Table)). Heat shocks (HS) were applied during the lag-phase (HS1), during the grain filling-phase (HS2) or during both the lag-phase and filling-phase (HS12) of the grain development, compared to Control.
(TIF)

S1 Table Some characteristics of the plant canopy at anthesis in the four containers selected for heat treatments. When available data are means ± 1SD. Within each column, means followed by the same letter were not different at a 5% level of significance (SNK test).
(DOCX)

S2 Table Type of growth function used to fit observations from anthesis to maturity for each trait. Equation and coefficient of determination (*R*^2^) for the fits between observations and predictions from non-linear mixed models for each trait and all temperature treatments.
(DOCX)

S3 Table Mean daily grain temperature (m ± SD, n = 4) over the two heat shock treatment periods (four consecutive days during lag-phase and/or filling phase of grain growth). The required mean daily air temperature was 18.5°C and 23.8°C under Control and heat shock treatments respectively.
(DOCX)

S4 Table Fresh and dry masses and volumes of the endosperm and OLs at maturity for wheat plants exposed to heat shocks. Heat shocks were applied during the lag-phase (HS1), the filling-phase (HS2) or both phases (HS12). For OLs, it was not possible to measure the volume as grains were dissected. Data are means ± 1SD (n = 5). Within each column for a given developmental stage, means followed by different letters were different.
(DOCX)
