# Supplementary figures and images for: Heat shock exposure during early wheat grain development can reduce maximum endosperm cell number but not necessarily final grain dry mass
Source: PLoS One. 2023 Apr 28;18(4):e0285218. doi: 10.1371/journal.pone.0285218 (PMC10146457; doi:10.1371/journal.pone.0285218)

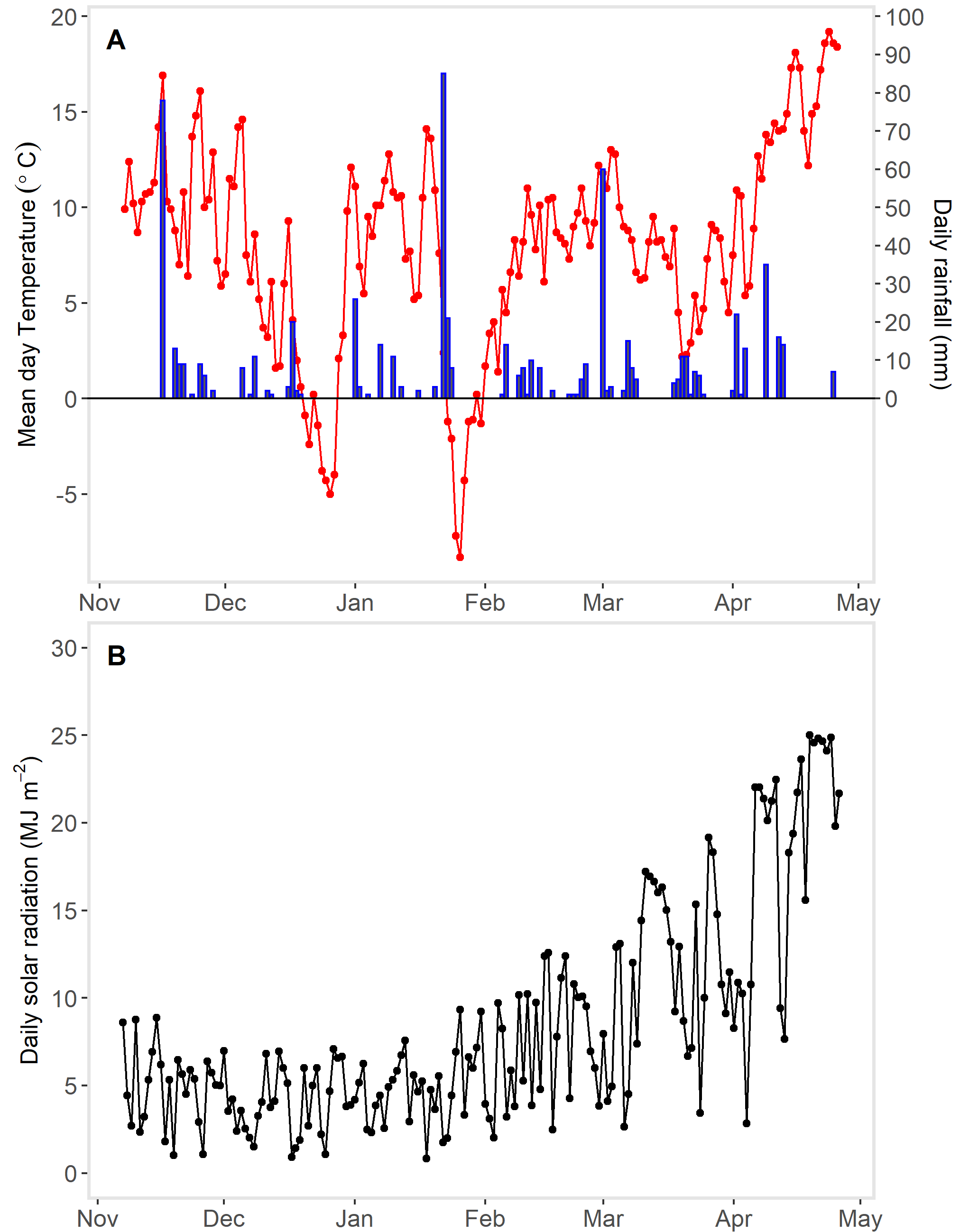

Supplement: S1 Fig — Mean day temperature (°CDAA) and daily rainfall (mm) (A) and daily solar radiation (MJ m-2) (B). (TIF) [file pone.0285218.s001.tif]

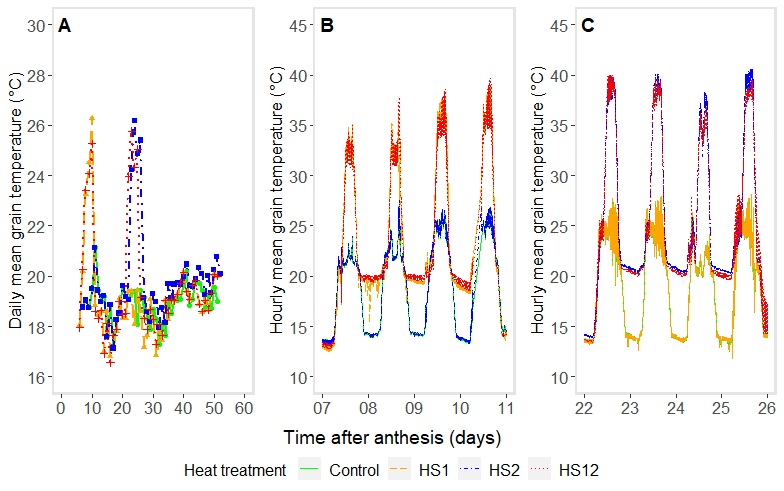

Supplement: S2 Fig — Daily mean grain temperature (A) over time from anthesis to grain maturity and hourly mean grain temperature over the four days of exposure during the first heat shock (lag-phase) (B) or during the second heat shock (filling-phase) (C). (TIF) [file pone.0285218.s002.tif]

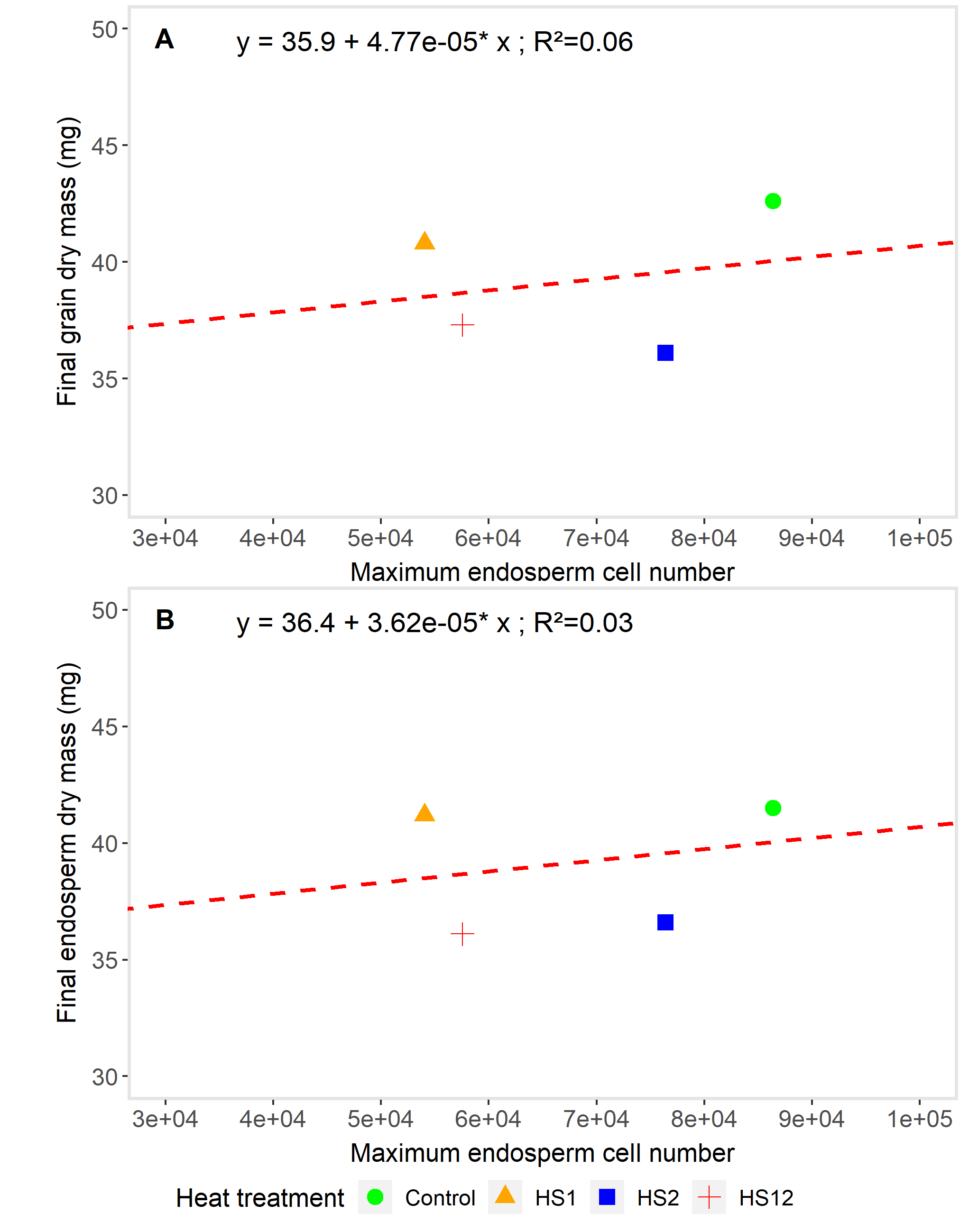

Supplement: S3 Fig — For each heat shock treatment, each point corresponds to the estimated mean value by the fitting of growth functions to the observed values (3-parms logistic and Gompertz with maxima for dry mass and endosperm cell number, respectively (S1 Table)). Heat shocks (HS) were applied during the lag-phase (HS1), during the grain filling-phase (HS2) or during both the lag-phase and filling-phase (HS12) of the grain development, compared to Control. (TIF) [file pone.0285218.s003.tif]

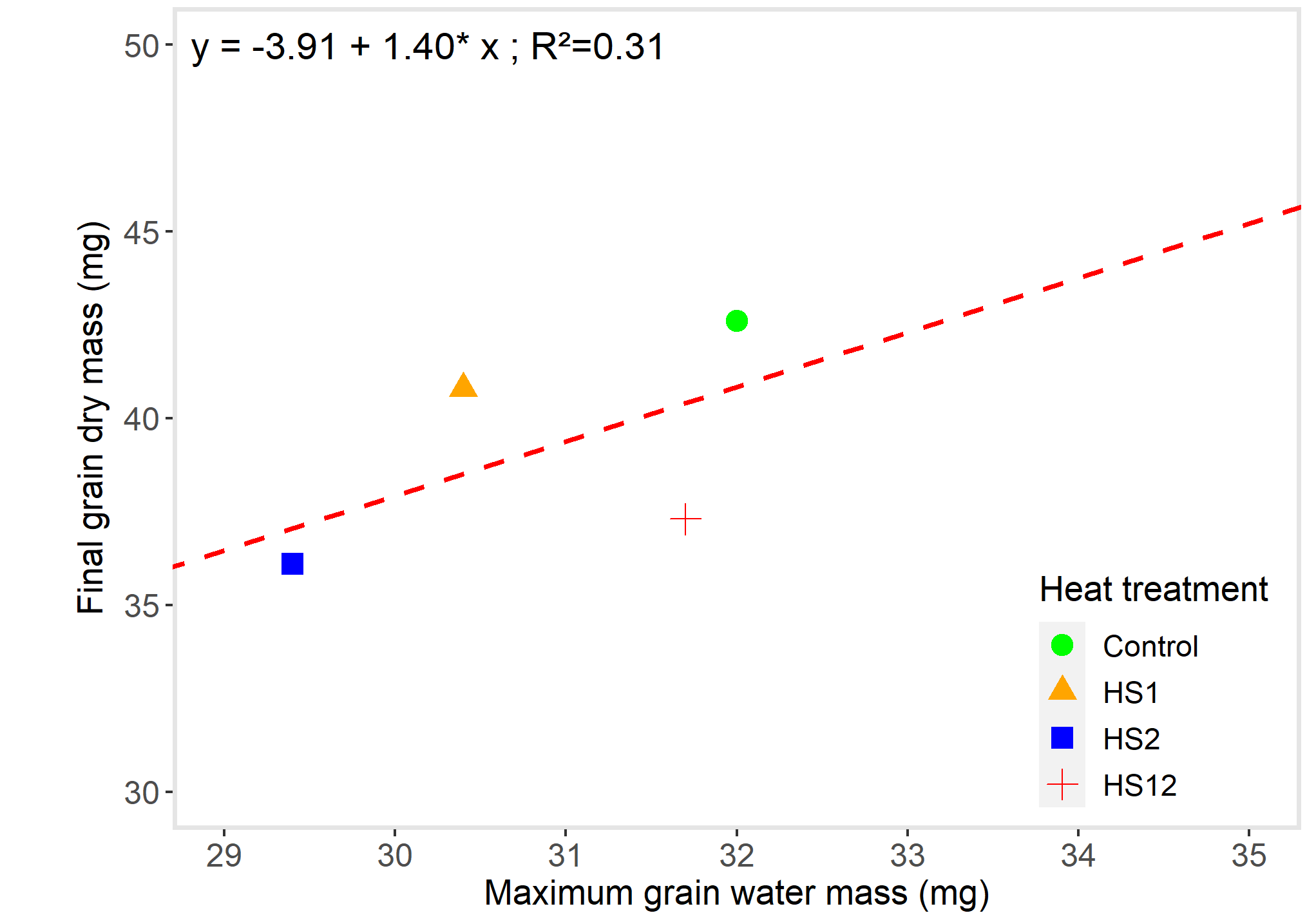

Supplement: S4 Fig — For each temperature treatment, each point corresponds to the estimated mean value by the fitting of growth functions to the observed values (3-parms logistic and segmented linear function for grain dry mass and water mass, respectively (S1 Table)). Heat shocks (HS) were applied during the lag-phase (HS1), during the grain filling-phase (HS2) or during both the lag-phase and filling-phase (HS12) of the grain development, compared to Control. (TIF) [file pone.0285218.s004.tif]
