# Supplementary material for: Heat shock exposure during early wheat grain development can reduce maximum endosperm cell number but not necessarily final grain dry mass
Source: PLoS One. 2023 Apr 28;18(4):e0285218. doi: 10.1371/journal.pone.0285218 (PMC10146457; doi:10.1371/journal.pone.0285218)
Supplement: S1 Table — When available data are means ± 1SD. Within each column, means followed by the same letter were not different at a 5% level of significance (SNK test). (DOCX) [file pone.0285218.s005.docx]

**S1 Table. Some characteristics of the plant canopies at anthesis in the four containers selected for heat treatments.** When available, data are means ± 1SD*.*  Within each column, means followed by the same letter were not different at a 5% level of significance (SNK test).

| **Container associated with treatment** | **Flowering date***  **(Julian date)** | **Spike length (mm)**** | **Spike mass**  **(g)**** | **Number of spikelets per spike***** | **Mean plant height**  **(cm)** |
| --- | --- | --- | --- | --- | --- |
| **C** | 119 | 81.4 ± 2.1 **a** | 1.28 ± 0.11 **a** | 18.8 ± 0.5 **a** | 88 |
| **HS1** | 120 | 77.0 ± 3.8 **a** | 1.16 ± 0.16 **a** | 18.4 ± 0.5 **a** | 79 |
| **HS2** | 119 | 80.0 ± 4.9 **a** | 1.13 ± 0.09 **a** | 19.0 ± 0.0 **a** | 80 |
| **HS12** | 120 | 83.2 ± 1.3 **a** | 1.25 ± 0.08 **a** | 18.8 ± 0.4 **a** | 85 |

*: The flowering date corresponds to the date when more than 50% of the spikes were at first anthesis stage. For all containers, flowering took place over 4 days (between days 118-121). The flowering date has been scored using 200 randomly the spikes of main stems.

**: The spike length and spike mass have been measured on 10 randomly sampled spikes of the main stems.

***: The number of spikelets per spike has been scored on 200 randomly spikes of the main stems.
