# Supplementary material for: Heat shock exposure during early wheat grain development can reduce maximum endosperm cell number but not necessarily final grain dry mass
Source: PLoS One. 2023 Apr 28;18(4):e0285218. doi: 10.1371/journal.pone.0285218 (PMC10146457; doi:10.1371/journal.pone.0285218)
Supplement: S2 Table — Equation and coefficient of determination (R2) for the fits between observations and predictions from non-linear mixed models for each trait and all temperature treatments. (DOCX) [file pone.0285218.s006.docx]

**S2 Table**. **Type of growth function used to fit observations from anthesis to maturity for each trait**. Equation and coefficient of determination (*R*^2^) for the fits between observations and predictions from non-linear mixed models for each trait and all temperature treatments.

| **Trait** | **Type of function** | **Observed-predicted relationship** | **R^2^** |
| --- | --- | --- | --- |
| **Grain fresh mass** | Gompertz with maxima^a^ | 3.27 + 0.91 x | 0.92 |
| **Grain dry mass** | 3-parms logistic^b^ | 0.42 + 0.96 x | 0.97 |
| **Grain volume** | Gompertz with maxima | 4.23 + 0.91 x | 0.91 |
| **Grain water mass** | Segmented linear function^c^ | 1.70 + 0.91 x | 0.91 |
| **Grain length** | Segmented linear function | 0.43 + 0.92 x | 0.92 |
| **Grain width** | Gompertz with maxima | 0.85 + 0.76 x | 0.76 |
| **OLs fresh mass** | Gompertz with maxima | 0.42 + 0.91 x | 0.84 |
| **OLs dry mass** | Gompertz with maxima | 0.45 + 0.81 x | 0.73 |
| **Endosperm cell number** | Gompertz with maxima | 1632 + 0.94 x | 0.93 |

^a^ Werker and Jaggard (1997) [59]

^b^  Triboi *et al.* (2003) [53]

^c^ Pepler *et al.* (2006) [58]
