# Supplementary material for: Heat shock exposure during early wheat grain development can reduce maximum endosperm cell number but not necessarily final grain dry mass
Source: PLoS One. 2023 Apr 28;18(4):e0285218. doi: 10.1371/journal.pone.0285218 (PMC10146457; doi:10.1371/journal.pone.0285218)
Supplement: S3 Table — The required mean daily air temperature was 18.5°C and 23.8°C under Control and heat shock treatments respectively. (DOCX) [file pone.0285218.s007.docx]

**S3 Table**. **Mean daily grain temperature (m ± SD, n=4) over the two HS treatment periods** (4 consecutive days during lag-phase and/or filling phase of grain growth). The required mean daily air temperature was 18.5°C and 23.8°C under Control and HS treatments respectively.

| **Temperature treatment** | **Lag-phase** | **Filling-phase** |
| --- | --- | --- |
| **Control** | 19.6 ± 1.1 | 19.1 ± 0.7 |
| **HS1** | 23.6 ± 2.6 | 19.1 ± 0.7 |
| **HS2** | 19.6 ± 1.1 | 25.2 ± 0.8 |
| **HS12** | 23.3 ± 2.1 | 24.8 ± 0.8 |
