# Supplementary material for: Heat shock exposure during early wheat grain development can reduce maximum endosperm cell number but not necessarily final grain dry mass
Source: PLoS One. 2023 Apr 28;18(4):e0285218. doi: 10.1371/journal.pone.0285218 (PMC10146457; doi:10.1371/journal.pone.0285218)
Supplement: S4 Table — Heat shocks were applied during the lag-phase (HS1), the filling-phase (HS2) or both phases (HS12). For OLs, it was not possible to measure the volume as grains were dissected. Data are means ± 1SD (n = 5). Within each column for a given developmental stage, means followed by different letters were different. (DOCX) [file pone.0285218.s008.docx]

**S3 Table**. **Fresh and dry masses, and volumes of the endosperm and OLs at maturity for wheat plants exposed to heat shocks.** Heat shocks were applied during the lag-phase (HS1), the filling-phase (HS2) or both phases (HS12). For OLs, it was not possible to measure the volume as grains were dissected. Data are means ± 1SD (n=5). Within each column for a given developmental stage, means followed by different letters were different at a 5% level of significance (SNK test).

|  | **Endosperm** | | |  | **OLs** | |
| --- | --- | --- | --- | --- | --- | --- |
| **Treatment** | **Fresh mass**  (mg grain^-1^) | **Dry mass**  (mg grain^-1^) | **Volume**  (mm^3^ grain^-1^) |  | **Fresh mass**  (mg grain^-1^) | **Dry mass**  (mg grain^-1^) |
| **Control** | 42.9 ± 7.9 **a** | 41.5 ± 4.7 **a** | 52.8 ± 7.8 **a** |  | 1.8 ± 0.3 **a** | 1.2 ± 0.1 **a** |
| **HS1** | 43.1 ± 7.3 **a** | 40.9 ± 7.7 **a** | 52.4 ± 4.3 **a** |  | 1.6 ± 0.4 a**b** | 1.0 ± 0.2 **ab** |
| **HS2** | 37.6 ± 7.3 **a** | 34.9 ± 4.4 **b** | 51.2 ± 6.5 **a** |  | 1.3 ± 0.2 **bc** | 1.0± 0.2 **ab** |
| **HS12** | 39.9 ± 3.4 **a** | 35.4 ± 4.4 **b** | 50.7 ± 2.5 **a** |  | 1.1 ± 0.1 **c** | 0.9 ± 0.1 **b** |
